# Advances in Induced Pluripotent Stem Cell Technologies

**DOI:** 10.1155/2012/850201

**Published:** 2012-06-19

**Authors:** Rajarshi Pal, Mahendra Rao, Mohan C. Vemuri, Paul Verma, Andras Dinnyes

**Affiliations:** ^1^Manipal Institute of Regenerative Medicine, Manipal University Branch Campus, Bangalore 560 071, India; ^2^NIH Center for Regenerative Medicine, Bethesda, MD 20892, USA; ^3^Life Technologies, Frederick, MD 21704, USA; ^4^Centre for Reproduction and Development, Monash Institute of Medical Research, Monash University, Clayton, VIC 33168, Australia; ^5^BioTalentum Ltd., Godollo 2100, Hungary

Progress in studying induced pluripotent stem cell (iPSC) has been extremely rapid. Ever since the remarkable discovery of iPSCs by Takahashi and Yamanaka, the field has continued to evolve with exciting discoveries furthering our understanding of early development, the process of cellular reprogramming, acquisition and maintenance of pluripotency, determination of cell fate, and enhancing our ability to model diseases in vitro. These advances and the possibility of generating patient or disease specific pluripotent cells placed the field on a trajectory that may lead to personalized cell therapy in the near future.

Although there have been significant advances in iPSC-based research, significant challenges remain and these will need to be addressed before tangible outcomes can be realized. Such challenges include developing robust strategies to reprogram cells free of a viral/genetic foot print, resolving the immune and potential tumorigenicity issues, understanding better the genome and epigenome status of iPSC, and developing techniques to predict the quality of iPSC clones. Understanding the basic biology of iPSCs well enough to allow for successful transition of iPSCs into the clinic someday will also require additional studies. 

To further discussion of the barriers in the field of iPSC biology, the guest editors of this special issue have compiled select original research findings and review articles describing important issues in iPSC research. These include manuscripts that describe the role of recombinant Sox-2 (M. Thier et al.) and cMyc (C. Heffernan et al.) protines in reprogramming of fibroblasts. Other manuscripts describe the development of better methods of iPSC derivation such as the use of small molecules (S. K. Mak et al.), HDAC inhibitors (A. Kretsovali et al.), nonintegrating sendai virus-based generation (C. C. MacArthur et al.), microRNA mediated reprogramming (C.-H. Kuo and S.-Y. Ying). Addition articles on developing iPSC lines from nonhuman primates (Y. Wu et al.), and other large animal models such as equines (K. Khodadadi et al.) highlight the importance of this aspect in translational efforts. Yet other papers deal with the prospects of iPSC-based genetic repair (J. A. Pawitan), techniques for high-resolution genomic profiling (K. H. Elliott et al.), the promise of iPSC in dental problems (T. C. Srijaya et al.), using iPSCs for drug discovery and for developing models of toxicity (R. S. Deshmukh et al.). Elliot et al. describe resolution genome profiling that will facilitate assessment of the quality of iPSC.

We are enthused to see the rapidity with which the field has advanced and how international in scope the field has become. It was gratifying for us to see papers from Asia, Europe, Australia, and America and it is perhaps appropriate given the title of the journal Stem Cells International.

We hope these latest review articles and original research articles in the field of iPSC research will promote and enable better understanding of the basic biology of iPSC that is critical for successful translation of iPSC for patient specific cell therapies in future.

*Rajarshi Pal* *Rajarshi Pal* *Mahendra Rao* *Mahendra Rao* *Mohan C. Vemuri* *Mohan C. Vemuri* *Paul Verma* *Paul Verma* *Andras Dinnyes* *Andras Dinnyes*

